# A Remarkable Clinical Test Described

**Published:** 1920-05-22

**Authors:** 


### THE WEIL-FELIX REACTION.

### A Remarkable Clinical Test Described.

The diagnosis of disease by bacteriological methods—or perhaps it would be better to say the confirmation of diagnosis—is a subject on which there are still many schools of thought. The Wassermann and the Widal reactions, the complement fixation test for the gonococcus and for hydatids, the skin reactions of Von Pirquet and Noguchi, to mention some of the best-known types, have all their supporters and detractors. But as knowledge increases, so the place these reactions hold in the physician's equipment becomes stronger and stronger. We can all remember the time when, should the result of a Wassermann reaction not fit in with the clinician's preconceived idea, that result was scouted; now we find it is frequently the case that the physician recasts his ideas to fit in with the Wassermann. In fact, subject to certain fairly definite limitations, one anticipates at least 99 per cent. direct hits from these laboratory methods.

### A New Test.

And now a new test appears, and one that cannot so easily be explained on theoretical grounds. Weil and Felix in 1915, working on a series of typhoid-fever-like cases in Galicia, failed to recover any organisms of the enteric group from either the blood, the fæces, or the urine of their patients. From the urine of one case, however, a typical organism of the proteus group was obtained, and it was found that this organism, which was for convenience described as $X_2$, was agglutinated by the patient's blood in dilutions as high as 1: 200. Later these cases were diagnosed typhus on clinical grounds and as a result of animal injections. This work, published in 1916, is the starting-point of this reaction.

Later a second organism, $X_{19}$, was isolated, which was more sensitive than $X_2$. Both these organisms are short, thin, slightly motile, Gram negative bacilli, and resemble *B. proteus*. On Conradi-Drigalski medium they give blue colonies, and on Endo-medium colourless, and later red colonies. The sugar reactions are those of the proteus group, but in the hands of different observers slightly different results have been obtained from well authenticated strains of $X_2$ and $X_{19}$.

### Detailed Characters.

Weil and Felix report the production of acid in glucose and lactose. Fairley reports the formation of acid and gas in glucose, saccharose, maltose, and mannite, and none in dulcite or lactose. Bengtson, in the United States, reports the formation of acid and gas in glucose, saccharose, and maltose, but none in lactose, mannite, or raffenose. All workers agree that indol is formed and gelatin is liquefied.

As to the agglutination reactions, the organisms $X_2$ and $X_{19}$ take their place in the proteus group in a very definite way—in fact, they serve as a standard by which this very difficult group of organisms may be classified. The whole group may be divided into three classes—those which are not agglutinated by sera sensitised against $X_2$ or $X_{19}$, those which are slightly agglutinated, and those which are strongly agglutinated. Not only are certain strains of *B. proteus* agglutinated by immune sera from $X_2$ and $X_{19}$, but also those two special strains may be agglutinated by sera from the corresponding strains of *B. proteus*. As to other serum reactions, complement fixation is negative, and the presence of precipitation is not proved.

### The Possible Mechanism.

The *raison d'être* of the test is by no means clear. Why this organism should bear any relation to typhus and what that relation is are almost purely matters of speculation. That they are the causal organism is not even suggested, for, though the patient's serum will constantly agglutinate the organism, the organism is by no means constantly found in the patient. Nor does inoculation either into the lower animals or man cause any of the symptoms of typhus. That these organisms are of the nature of secondary invaders is also negatived by the same fact that the occurrence of either $X_2$ or $X_{19}$ is not a constant occurrence in typhus. The absence of complement fixation, too, is against this, especially as inoculation with a vaccine prepared from these organisms produced in the subject of the experiment both agglutinating powers and an immune body for complement fixation.

### Another Theory.

Another theory, and one which certainly seems to fit in with the known facts of the case, is that in typhus large quantities of agglutinins are formed. Witness the literature on the subject, which records that *B. typhi-exanthematica* of Ploty, *B. Typhosus*, and *Bacillus* "*U*" of Wilson, though vastly different organisms, have all been agglutinated by the sera of typhus patients. These agglutinins are not those primarily concerned with the causal organism, but secondary, almost accidental bodies which happen to act on these organisms. That this is not an entirely unreasonable idea is suggested by the nature of the syphilitic antigen in the Wassermann reaction. This, to begin with, was an alcoholic extract of syphilitic fœtal liver, a substance known to be rich in the pale spirochæte, while nowadays normal heart extract with an alcoholic solution of cholesterol is almost always used, which would lead one to believe that not any component of the cell substance of the spirochæte

**The Weil-Felix Reaction**—(*continued*).

itself, but some lipoid body produced by the degeneration of the cells of the patient, was what was needed.

### The Clinical Value.

The value of the reaction may be judged from the figures published by Fairley of results obtained by him in Egypt. Out of sixty-five cases diagnosed as typhus on clinical grounds sixty-three, or 97 per cent., gave a positive result; while out of 120 non-typhus patients none gave any agglutination, even in dilutions of 1 in 20. Weil and Felix report 125 positive reactions out of 126 cases of clinical typhus, while in 632 cases of other diseases 12 per cent. gave, in dilutions of 1 in 25, a delayed incomplete agglutination.

The reaction is not constantly present till after the fifth or seventh day, but once present it continues for some weeks. In a few very mild and very severe cases the reaction failed.

The technique of Weil and Felix is as follows: A twenty-four-hour culture of $X_{19}$ on agar is suspended in isotonic saline, and in early cases—*i.e.*, sera collected up to the end of the first week—is tested against dilutions of 1 in 25 and 1 in 50 of the suspected sera. Typhus cases should give a positive result in 1 in 25. On about the twelfth to fourteenth day a second test is made with the serum diluted 1 in 100 to 1 in 500. If the titre of the serum is not increased typhus may usually be excluded, whereas in typhus cases the titre is raised from 1 in 25 to 1-200, or even more. Some observers recommend killed organisms instead of fresh emulsions, others recommend heating the culture to 60° C. to increase its agglutinability. Alcoholic suspensions are advised by others as being very stable and reliable.

### Conclusions Drawn.

A test which is reliable in 97 to 99 per cent. of cases, and which is not affected by protective inoculation, as is the Widal reaction, is of the utmost value not only in countries like Great Britain, where, owing to its rarity, typhus is seldom seen and when seen is often not diagnosed, but particularly in those countries such as South Russia and the Balkans, where the differential diagnosis of typhus, typhoid, and relapsing fever may be a daily affair, and where the rapid diagnosis of early cases may prove the vital means of nipping a potential epidemic in the bud.

